# Polyclonal Immunoglobulin Recovery after Autologous Stem Cell Transplantation Is an Independent Prognostic Factor for Survival Outcome in Patients with Multiple Myeloma

**DOI:** 10.3390/cancers12010012

**Published:** 2019-12-18

**Authors:** Shuji Ozaki, Takeshi Harada, Hikaru Yagi, Etsuko Sekimoto, Hironobu Shibata, Toshio Shigekiyo, Shiro Fujii, Shingen Nakamura, Hirokazu Miki, Kumiko Kagawa, Masahiro Abe

**Affiliations:** 1 Department of Hematology, Tokushima Prefectural Central Hospital, Tokushima 770-8539, Japan; 2 Department of Hematology, Tokushima University Hospital, Tokushima 770-8503, Japan

**Keywords:** multiple myeloma, autologous stem cell transplantation, immunoglobulin recovery

## Abstract

We retrospectively analyzed multiple myeloma (MM) patients who underwent autologous stem cell transplantation (ASCT) without maintenance therapy to assess the impact of recovery of normal immunoglobulin (Ig) on clinical outcomes. The recovery of polyclonal Ig was defined as normalization of all values of serum IgG, IgA, and IgM 1 year after ASCT. Among 50 patients, 26 patients showed polyclonal Ig recovery; 14 patients were in ≥complete response (CR) and 12 remained in non-CR after ASCT. The patients with Ig recovery exhibited a significantly better progression-free survival (PFS, median, 46.8 vs. 26.7 months, *p* = 0.0071) and overall survival (OS, median, not reached vs. 65.3 months, *p* < 0.00001) compared with those without Ig recovery. The survival benefits of Ig recovery were similarly observed in ≥CR patients (median OS, not reached vs. 80.5 months, *p* = 0.061) and non-CR patients (median OS, not reached vs. 53.2 months, *p* = 0.00016). Multivariate analysis revealed that non-CR and not all Ig recovery were independent prognostic factors for PFS (HR, 4.284, 95%CI (1.868–9.826), *p* = 0.00059; and HR, 2.804, 95%CI (1.334–5.896), *p* = 0.0065, respectively) and also for OS (HR, 8.245, 95%CI (1.528–44.47), *p* = 0.014; and HR, 36.55, 95%CI (3.942–338.8), *p* = 0.0015, respectively). Therefore, in addition to the depth of response, the recovery of polyclonal Ig after ASCT is a useful indicator especially for long-term outcome and might be considered to prevent overtreatment with maintenance therapy in transplanted patients with MM.

## 1. Introduction

Multiple myeloma (MM) is a plasma cell neoplasm characterized by the presence of monoclonal immunoglobulin (Ig) in serum and/or urine and clinical symptoms related to hypercalcemia, renal insufficiency, anemia, and bone lesion (CRAB features), and myeloma-defining events [1,2]. MM is a clinically and cytogenetically heterogeneous disease, and survival outcome varies considerably depending on the risk status such as disease stage and cytogenetic abnormalities, and treatment of each patient [3].

Until now, treatment strategy for MM has been evolving rapidly by the introduction of several new classes of agents such as proteasome inhibitors (bortezomib, carfilzomib, and ixazomib), immunomodulatory drugs (thalidomide, lenalidomide, and pomalidomide), and monoclonal antibodies (elotuzumab and daratumumab) [4]. These highly effective modalities including bortezomib + lenalidomide + dexamethasone (VRd) induction and autologous stem cell transplantation (ASCT) have led to durable and deeper responses [5]. Some patients have achieved minimal residual disease (MRD) negativity that is now considered as a major prognostic factor for progression-free survival (PFS) and overall survival (OS) [6,7]. In addition, randomized clinical trials have shown the efficacy of continuous therapy in non-transplanted patients and maintenance therapy after ASCT in transplanted patients for both PFS and OS [8,9]. However, adverse events such as second primary malignancies and infections have been reported with lenalidomide maintenance [9,10], and gastrointestinal disorders and rash with ixazomib maintenance [11]. Therefore, it has not been established whether patients with deep response such as MRD negative have real benefit from continuous and maintenance therapy because of possible adverse events and cost problems in routine clinical practice [12,13].

On the other hand, most patients present with immunoparesis at diagnosis, which is shown to be another important prognostic factor associated with PFS and OS [14,15,16]. Since subsequent treatment itself induces humoral and cellular immunodeficiency, the balance between treatment intensity and immune recovery is a crucial issue for improving the long-term disease stability. Several studies have reported that patient immunity assessed by serum Ig levels and lymphocyte counts in the peripheral blood is associated with prognosis, and immune recovery after successful treatment is significantly related to a favorable outcome [17,18,19,20,21]. In addition, recent studies have shown that a favorable immune signature including B cells, T cells, and NK cells as well as humoral immunity exerts a competent anti-tumor immune surveillance, which results in longer survival after ASCT [20,21]. Therefore, it is necessary to evaluate both the depth of response and the recovery of immunity in considering the optimal treatment strategy for the long-term outcome in individual patients.

Recently, polyclonal Ig recovery after ASCT has been shown to be a favorable prognostic factor for PFS and OS [17,18]. However, in these studies approximately 80% of patients were under maintenance therapy such as interferon-α and lenalidomide, and neither the significance of immune recovery after ASCT alone nor the true effectiveness of maintenance therapy has been clarified. In the present study, we have evaluated the polyclonal Ig recovery after ASCT as a simple indicator and compared the impact on survival outcome with therapeutic response in transplanted patients who did not receive maintenance therapy.

## 2. Results

### 2.1. Patients’ Characteristics

A total of 50 patients (23 male and 27 female) were included in this study (Table 1). The median age was 57 (range, 35–71) years. The type of monoclonal Ig was 24 IgG, 9 IgA, 2 IgD, 13 light-chain only, and 2 non-secretory. At diagnosis, anemia (hemoglobin < 10 g/dL), renal failure (serum creatinine > 2.0 mg/dL), and hypercalcemia (serum calcium >11 mg/dL) were observed in 42%, 5%, and 13% of patients, respectively. Extensive bone destruction in 3 or more lesions was observed in 50% of patients. The International Staging System (ISS) stages I, II, and III were 16, 20, and 13 (1 unknown); and the Revised International Staging System (R-ISS) stages were 9, 25, and 5 (11 unknown), respectively. All patients received upfront ASCT after induction therapy either vincristine + doxorubicin + dexamethasone (VAD, *n* = 20) or novel agent-based therapy such as bortezomib + dexamethasone (BD, *n* = 30). As for the therapeutic response, 5 patients achieved stringent complete response (sCR), 6 CR, 11 very good partial response (VGPR), 24 PR, and 4 stable disease (SD) after induction therapy. Fourteen patients achieved sCR, 7 CR, 14 VGPR, 13 PR, and 2 SD as best response after ASCT.

### 2.2. Polyclonal Ig Recovery after ASCT

Serum levels of IgG, IgA, and IgM were measured 1 year after ASCT, and the recovery of polyclonal Ig was defined as normalization of all values of IgG, IgA, and IgM. One year after ASCT, 26 patients (52%) showed all three types of Ig recovery, whereas 24 patients (48%) remained without all Ig recovery (6 patients achieved two types of Ig recovery, 8 one type of Ig recovery, and 10 none Ig recovery). Patients who did not recover all Igs tended to fail to recover IgA and/or IgM, and the differences by the class of Ig recovered could not be evaluated due to the small number.

The percentage of patients with all Ig recovery was not significantly different in age, gender, type of M protein, hemoglobin, serum creatinine, serum calcium, bone lesion, and the ISS stage (Table 1). Although the number of patients examined was small, the percentage of patients with all Ig recovery was more frequent in the stage I (8 of 9 patients, 89%) and less in the stage III (1 of 5 patients, 20%) according to the R-ISS classification (*p* = 0.036). FISH test was performed in 24 patients and 3 patients had the t(4;14) translocation (2 patients achieved all Ig recovery and 1 one type of Ig recovery). No patient had the t(14;16) translocation. Karyotype abnormality was observed in 4 patients (1 patient achieved all Ig recovery, 1 one type of Ig recovery, and 2 none Ig recovery).

In relation to treatment, the rate of Ig recovery was not significantly different by the type of induction regimen. Tandem ASCT was performed in 3 patients, and 1 achieved all Ig recovery, 1 two types of Ig recovery, and 1 none Ig recovery. According to the therapeutic response before ASCT, Ig recovery 1 year after ASCT was observed in 8 patients (73%) in the ≥CR group and 18 patients (46%) in the non-CR group. According to the therapeutic response after ASCT, Ig recovery was observed in 14 patients (67%) in the ≥CR group and 12 patients (44%) in the non-CR group. Thus, Ig recovery was more frequently observed in patients with deeper response, but there was no statistically significant difference between 2 groups such as the ≥CR group vs. the non-CR group (*p* = 0.18 and *p* = 0.093, respectively).

### 2.3. Polyclonal Ig Recovery and Bone Marrow Plasma Cells

Bone marrow examination was performed only in ≥CR patients at the time of evaluation of therapeutic response before and after ASCT, and the absence of monoclonal MM cells was confirmed by immunohistochemistry in these patients. In patients where the percentage of normal plasma cells was measured by aspiration, it ranged 0.1–6.4% (median, 1.0%; *n* = 6) before ASCT and 0.4–4.8% (median, 2.0%; *n* = 7) after ASCT, respectively. Bone marrow examination was not necessarily done 1 year after ASCT and the relationship between the percentage of normal plasma cells and Ig recovery could not be evaluated.

### 2.4. Survival Outcome

**Figure 1:**
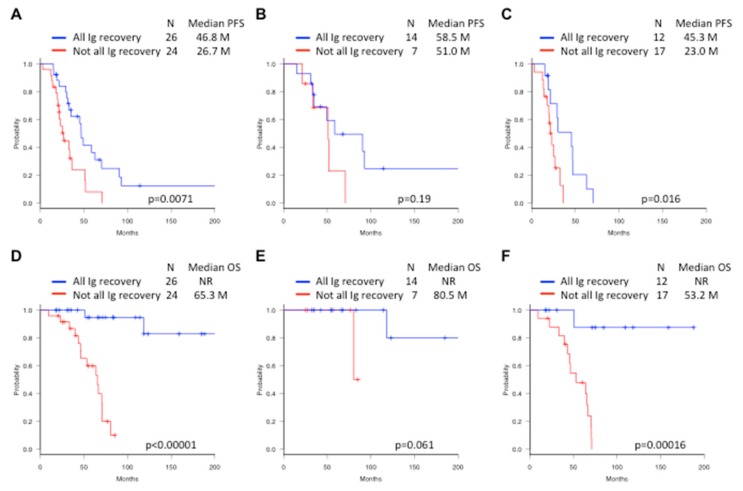
Progression-free survival (PFS; **A**, **B**, and **C**) and overall survival (OS; **D**, **E**, and **F**) according to the status of Ig recovery either all three Ig recovery or not all Ig recovery. Patients with all Ig recovery had improved PFS (**A**) and OS (**D**) compared with those without all Ig recovery. PFS and OS in ≥CR patients (**B** and **E**) and non-CR patients (**C** and **F**) by therapeutic response after ASCT. Time from induction therapy (months). NR, not reached.

The median PFS in all patients was 35.0 months. Notably, improvement of PFS was observed depending on the number of Ig recovery; the median PFS in none Ig recovery group was 21.4 months, one type was 23.0 months, two types was 36.0 months, and all three types was 46.8 months, respectively (*p* = 0.005). Thus, the patients who recovered all three Igs were the best and had a significantly better PFS than the patients who did not recover all Ig (median, 46.8 vs. 26.7 months, *p* = 0.0071, Figure 1A). When analyzed by treatment response, there was no significant difference in PFS between the all Ig recovered and not all recovered patients in the ≥CR group (*p* = 0.19, Figure 1B). In contrast, there was a significant difference in PFS between the all Ig recovered and not all recovered patients in the non-CR group (median, 45.3 vs. 23.0 months, *p* = 0.016, Figure 1C).

Subsequently, 18 of 26 patients (69%) with Ig recovery and 18 of 24 patients (75%) without Ig recovery relapsed during the median observation period of 63.9 months. The median OS in all patients was 118.3 months. Similarly, improvement of OS was observed depending on the number of Ig recovery; the median OS in none Ig recovery group was 53.2 months, one type was 63.8 months, two types was 68.1 months, and all three types was not reached, respectively (*p* = 0.000022). The patients with all Ig recovery had a significantly better OS compared with the patients without all Ig recovery (median, not reached vs. 65.3 months, *p* < 0.00001, Figure 1D). According to the treatment response, the survival benefits of Ig recovery were similarly observed in ≥CR patients (median OS, not reached vs. 80.5 months, *p* = 0.061, Figure 1E) and non-CR patients (median OS, not reached vs. 53.2 months, *p* = 0.00016, Figure 1F).

### 2.5. Univariate and Multivariate Analysis

To evaluate the significance of prognostic factors on PFS and OS, we performed univariate and multivariate analysis including the factors such as age (≥65 years), gender (male), ISS (stage III), induction regimen (novel agent-based therapy), best response after ASCT (non-CR), and Ig recovery (not all recovery). R-ISS was not included because of a small number of patients that could be examined. Univariate and multivariate analysis revealed that both non-CR and not all Ig recovery were significant poor prognostic factors for PFS, and non-CR was a more significant factor than not all Ig recovery by multivariate analysis (*p* = 0.00059 and *p* = 0.0065, respectively, Table 2). In regard to OS, both non-CR and not all Ig recovery were significant poor prognostic factors, and not all Ig recovery was a more significant factor than non-CR (*p* = 0.0015 and *p* = 0.014, respectively, Table 3).

## 3. Discussion

In the present study, we retrospectively analyzed the clinical relevance of Ig recovery in MM patients who underwent ASCT without maintenance as initial therapy. We have demonstrated that patients with polyclonal Ig recovery 1 year after ASCT had a significantly better PFS and OS than those without Ig recovery. Multivariate analysis revealed that recovery of all three Igs was an independent favorable prognostic factor for PFS and OS. Thus, the recovery of polyclonal Ig after ASCT is a clinically useful indicator for long-term outcome in patients with MM.

The polyclonal Ig recovery is thought to reflect the reconstitution of suppressed normal B-cell function. In this context, the emergence of oligoclonal bands was also considered to reflect the recovery of humoral immunity as a result of clonal competition between neoplastic plasma cells and normal plasma cells [22,23,24]. In particular, persistence of this humoral response for over 1 year after ASCT was associated with better PFS and OS, suggesting the importance of continuous immune recovery for long-term outcome [25]. In a kinetic study of polyclonal Ig recovery after ASCT, the number of patients with Ig recovery increased over time and reached the maximum at 1 year after ASCT, when B-cell reconstitution is expected to be completed [17]. Therefore, these results suggest that polyclonal Ig recovery 1 year after ASCT is a simple and reliable indicator for assessing immune recovery in transplanted patients with MM.

As an alternative method for monitoring M proteins and normal Ig, heavy + light chain (HLC) immunoassays have become available, which can quantify IgGκ/IgGλ, IgAκ/IgAλ, and IgMκ/IgM/λ separately [26]. Several studies have reported the usefulness of HLC assay for monitoring the concentration of M protein (involved) and polyclonal Ig (uninvolved) not only for precise measurement of M protein as assessment of response but also for surrogate marker of polyclonal Ig suppression and recovery (HLC-matched pair) [27,28]. Severe HLC-matched pair suppression at diagnosis was related to poor PFS and OS, and recovery of HLC ratio after ASCT was associated with longer OS [26,29,30,31]. Taken together, these results also suggest that the recovery of humoral immunity is profoundly related to favorable prognosis in patients with MM.

In the relationship between polyclonal Ig recovery and therapeutic response, Ig recovery 1 year after ASCT was more frequently observed in patients who reached ≥CR before and after ASCT than in non-CR patients, suggesting that Ig recovery is affected by the amount of remaining tumors that suppress normal plasma cells. With regard to risk factors, polyclonal Ig recovery was mostly observed in R-ISS stage I patients but less in R-ISS stage III. The reason for this might be that the patients with R-ISS stage I responded well and most of them achieved CR and subsequent Ig recovery, while patients with R-ISS stage III or high risk may suffered a long time enough to cause clonal evolution and normal plasma cells might be more strongly suppressed, and thus humoral immune recovery was more difficult to occur. As support for this hypothesis, several studies have reported that patients with R-ISS stage III and adverse cytogenetic abnormalities were more likely to have suppression of Ig at diagnosis [15,16]. Further studies are needed to elucidate the mechanism of immunosuppression and the relationship between cytogenetic abnormality of MM cells and its inhibitory effects on normal plasma cells.

In terms of PFS and OS, multivariate analysis have shown that the depth of response shown by ≥CR was more significantly associated with the short-term disease stability such as PFS, whereas immune recovery shown by polyclonal Ig recovery was more significantly associated with OS that is a marker of long-term survival including after relapse. Notably, the survival benefits of Ig recovery were similarly seen in both ≥CR (Figure 1E) and non-CR (Figure 1F) groups. Non-CR patients with Ig recovery could be considered as if they had returned into monoclonal gammopathy of undetermined significance (MGUS)-like status that do not achieve CR but have an excellent outcome [32]. Therefore, it can be speculated that Ig recovery as a part of immune reconstitution contributes to disease stability in non-CR patients or even after relapse irrespective of the presence of residual disease, and appears to be a more powerful indicator for long-term outcome than ≥CR response.

Because the treatment itself contributes to immunosuppression in MM, certain types of therapeutic agents may affect the potential of polyclonal Ig recovery. Proteasome inhibitors such as bortezomib and carfilzomib strongly inhibit antibody-producing plasma cells and may reduce the normal Ig levels [33] as evidenced by the high incidence of varicella-zoster virus reactivation in patients treated with bortezomib [34]. In contrast, immunomodulatory drugs such as lenalidomide and pomalidomide enhance cellular immunity through Th1 cytokine production [35]. In fact, uninvolved Ig recovery was observed in MM patients treated with long-term lenalidomide therapy either as salvage or maintenance settings [36,37]. Moreover, anti-CD38 monoclonal antibody, daratumumab, has been shown to deplete CD38+ immunosuppressive cells and modulate T-cell functional response although the clinical relevance remains to be elucidated [38]. Because these key drugs might affect immune function and the normal Ig levels, it might be difficult to assess Ig recovery under the current continuous or maintenance therapy.

The present study has several limitations because of its retrospective nature and relatively small sample size. First, we did not perform MRD assay because the assay was not available at that time, and were unable to evaluate the clinical significance of Ig recovery in comparison with MRD negativity that is one of the most powerful prognostic factors [6,7]. Second, induction therapy was heterogeneous in VAD or bortezomib-based therapy but not VRd, the current standard therapy. Although there was no significant difference by the type of induction regimen in the Ig recovery rate after ASCT, initial therapy might be important because deeper response after induction therapy tended to contribute to more frequent Ig recovery. Third, maintenance therapy was not performed in these patients, and the results of this study cannot be interpreted with current patients on maintenance therapy. Previous studies have reported that Ig recovery 1 year after ASCT was a favorable prognostic factor for PFS and OS even in patients undergoing maintenance therapy [17,18]. However, if patients were in CR or MRD negative after ASCT, it might be possible to suspend maintenance therapy to confirm immune recovery, which may contribute to preventing overtreatment and possible adverse events.

## 4. Materials and Methods

### 4.1. Patients and Treatment

Between 1994 and 2017, 50 newly diagnosed patients received upfront ASCT and followed up at our hospitals. The diagnosis of MM was made according to the IMWG criteria [1,2]. Bone lesions at diagnosis were examined by skeletal radiography or computed tomography, and the disease severity was defined by the number of osteolytic lesions (0, 1–3, and >3). The ISS and the R-ISS stages in each patient were determined according to the respective IMWG criteria previously published [39,40].

Patients were treated with VAD or novel agent-based therapy such as BD as induction and subsequently treated with high-dose melphalan (200 mg/m^2^) followed by ASCT. Maintenance therapy was not performed after ASCT. Treatment after relapse was determined by the respective physician-in-charge. The follow-up period was 9.4–296.2 (median, 63.9) months.

The recovery of polyclonal Ig was defined as normalization of values of serum IgG, IgA, and IgM by routine laboratory examination 1 year after ASCT. The normal range used in our hospitals is based on the reference intervals proposed by the Japanese Committee for Clinical Laboratory Standards and is as follows: IgG, 861–1747 mg/dL; IgA, 93–393 mg/dL; and IgM, 33–183 mg/dL for male, 50–269 mg/dL for female [41]. Treatment response was assessed according to the uniform response criteria by the IMWG [42]. This study was conducted in accordance with the institutional guidelines with approval of the Ethics Committee/Institutional Review Board of Tokushima Prefectural Central Hospital (the ethical protocol number #19-12).

### 4.2. Statistical Analysis

Fisher’s exact test was used to compare differences between categorical variables, whereas the Mann-Whitney U test was used for continuous or nominal values. Kaplan–Meier method was used to create the OS curves, and differences between the curves were analyzed by the log-rank test. Cox model was used to estimate the hazard ratio with 95% confidence intervals (CI) in univariate and multivariate analysis on survival outcomes. Statistical analyses were performed with EZR version 1.30 (Saitama Medical Center, and Jichi Medical University, Saitama, Japan), which is a graphical user interface for R (The R Foundation for Statistical Computing, Vienna, Austria) [43].

## 5. Conclusions

In conclusion, our data demonstrates that patients with polyclonal Ig recovery 1 year after ASCT have a significantly longer PFS and OS compared with patients without Ig recovery even without maintenance therapy. The polyclonal Ig recovery is a simple and useful method to assess immune function and predict long-term outcome in addition to the depth of response. Thus, it might be necessary to consider the indication of further post-ASCT therapy individually depending on immune recovery to prevent overtreatment in transplanted patients with MM.

## Figures and Tables

**Table 1:** Patients’ characteristics.

Characteristic	All Ig recovery (*n* = 26)	Not all Ig recovery (*n* = 24)	Total (*n* = 50)	*p*
Median age (range)	58 (39–71) yrs	56 (35–69) yrs	57 (35–71) yrs	0.16
Gender (M/F)	11/15	12/12	23/27	0.78
M protein				0.89
IgG	11	13	24	
IgA	6	3	9	
IgD	1	1	2	
BJP	7	6	13	
Non-secretory	1	1	2	
Hemoglobin				0.74
Normal	12	10	22	
Low (<10g/dL)	7	9	16	
Unknown	7	5	12	
Serum creatinine				1.00
Normal	19	21	40	
High (>2mg/dL)	1	1	2	
Unknown	6	2	8	
Serum calcium				1.00
Normal	17	16	33	
High (>11mg/dL)	2	3	5	
Unknown	7	5	12	
Lytic bone lesion				0.20
0	4	8	12	
1–3	6	7	13	
>3	16	9	25	
ISS stage				0.48
I	10	6	16	
II	10	10	20	
III	5	8	13	
Unknown	1	0	1	
R-ISS stage				0.036
I	8	1	9	
II	13	12	25	
III	1	4	5	
Unknown	4	7	11	
Induction regimen				0.56
VAD	9	11	20	
Novel agent-based	17	13	30	
Response before ASCT				0.11
sCR	3	2	5	
CR	5	1	6	
VGPR	7	4	11	
PR	11	13	24	
SD	0	4	4	
Response after ASCT				0.36
sCR	9	5	14	
CR	5	2	7	
VGPR	5	9	14	
PR	7	6	13	
SD	0	2	2	

Ig: immunoglobulin, ISS: International Staging System; R-ISS: Revised International Staging System; VAD: vincristine + doxorubicin + dexamethasone. yrs: years.

**Table 2:** Univariate and multivariate analysis for progression-free survival.

Factors	Univariate		Multivariate	
	HR (95% CI)	*p*	HR (95% CI)	*p*
Age (≥65 yrs)	1.160 (0.467–2.880)	0.75	-	-
Gender (Male)	2.130 (1.036–4.381)	0.04	1.401 (0.668–2.936)	0.37
ISS (stage III)	1.636 (0.811–3.299)	0.17	-	-
Induction regimen (novel agent-based)	1.019 (0.523–1.986)	0.96	-	-
Response after ASCT (non-CR)	4.312 (2.000–9.295)	0.00019	4.284 (1.868–9.826)	0.00059
Ig recovery (not all)	2.533 (1.261–5.087)	0.009	2.804 (1.334–5.896)	0.0065

HR: hazard ratio; CI confidence interval; ISS: International Staging System; Ig: immunoglobulin.

**Table 3:** Univariate and multivariate analysis for overall survival.

Factors	Univariate		Multivariate	
	HR (95% CI)	*p*	HR (95% CI)	*p*
Age (≥65 yrs)	0.354 (0.0466–2.684)	0.31	-	-
Gender (Male)	1.409 (0.541–3.668)	0.48	-	-
ISS (stage III)	1.231 (0.454–3.340)	0.68	-	-
Induction regimen (novel agent-based)	0.358 (0.115–1.114)	0.076	-	-
Response after ASCT (non-CR)	7.595 (1.727–33.4)	0.0073	8.245 (1.528–44.47)	0.014
Ig recovery (not all)	29.46 (3.815–227.6)	0.0012	36.55 (3.942–338.8)	0.0015

HR: hazard ratio; CI confidence interval; ISS: International Staging System; Ig: immunoglobulin.
